# Fertility with early reduction of ovarian reserve: the last straw that breaks the Camel’s back

**DOI:** 10.1186/s40738-017-0041-1

**Published:** 2017-10-11

**Authors:** Sabahat Rasool, Duru Shah

**Affiliations:** 1 Gynaecworld, Kwality House, 1st Floor, Kemps Corner, Mumbai, India; 2 Scientific Director & Fertility Expert, Gynaecworld, Kwality House, 1st Floor, Kemps Corner, Mumbai, India

**Keywords:** Ovarian reserve, Diminished ovarian reserve, Reduced fertility, Poor ovarian response, Poor responders

## Abstract

Diminished fertility and poor ovarian response pose a conundrum to the experts in the field of reproductive medicine. There is limited knowledge about the risk factors of diminished ovarian reserve other than the iatrogenic ones. One of the leading causes of infertility in women today is diminished ovarian reserve (DOR). DOR is characterized by a low number of eggs in a woman’s ovaries and/or with poor quality of the remaining eggs, which boils down to impaired development of the existing eggs, even with assisted reproductive techniques. A good number of such women with low ovarian reserve may conceive with their own eggs, if they are given individualized treatment that is tailored for their profile. Such patients should be counseled appropriately for an aggressive approach towards achieving fertility. The sooner the treatment is started, the better the chances of pregnancy.

## Background

Little is known about the risks, and management of diminished ovarian reserve (DOR), which is affected by age, genetics and environmental factors. The one thing certain about DOR is that it is irreversible and that these women are at risk of poor ovarian response to ovarian stimulation in Assisted Reproductive Technologies (ART) [1, 2]. DOR is a poor prognostic factor in ART, because of a decline in the quantity and quality of oocyte [3]. Age is the most well – known contributing factor to DOR, and probably the most important prognostic factor in fertility treatment of women with DOR [3].Age-related abnormal vascularization, oxidative stress, free radical imbalance, toxic and genetic changes, all contribute to the declining oocyte quality, which translates into abnormal fertilization, and disordered embryo implantation [3]. DOR is associated with poor ovarian response to ovarian stimulation, higher cycle cancellation rates and lower pregnancy rates during In Vitro Fertilization (IVF) [4].

## Definition

DOR is defined as a decrease in the number of quality & quantity of oocytes [5]. It is used to describe women of reproductive age with regular cycles mostly ovulatory, whose response to stimulation or fecundity is reduced compared to women of comparable age. It is distinct from menopause or premature ovarian insufficiency. A woman’s chronological age is not the only determinant of ovarian reserve. Some studies have demonstrated an association between the cause of DOR and IVF outcome.

Poor ovarian response (POR) implies a subnormal follicular response, which means less number of eggs retrieved after ovarian stimulation during IVF [4]. ESHRE defined POR using Bologna criteria in order to standardize the definition, since the variability in the definition of POR was striking. According to Bologna criteria, POR is defined as “when at least two of the following three characteristics are present”:

1. Advanced maternal age > 40 yrs. or any other risk factors for poor ovarian response.
2. Previous POR (≤ 3 oocytes with conventional stimulation of >149 IU FSH daily), and
3. An abnormal ovarian reserve test (AFC < 5–7, or AMH < 0.5–1.1 ng/ml)

Two episodes of POR after maximal ovarian stimulation are sufficient to label a patient as a poor responder [6]. The most important reason for a poor ovarian response is DOR. Some subgroups of DOR, based on the cause of DOR, show better IVF outcome than others. A recent retrospective study showed better response to IVF in DOR caused by surgery for endometrioma compared to idiopathic group [7].

Yun H, et al. studied the IVF outcome of women with diminished ovarian reserve in 99 cycles and reported a clinical rate of 11.5% per cycle, cancellation rate of 34.4%. They found that DOR caused by previous ovarian surgery had better pregnancy outcome, while that caused by chemotherapy / gonadotoxic therapy had significantly higher cycle cancellation rate [4]. Studying the trends in DOR assignment in the Society for Assisted Reproductive Technology (SART) Clinic outcomes Reporting System Database and evaluating its accuracy for POR prediction, Devine K, et al. reported an increased prevalence of DOR from 19% to 26% from 2004 to 2011 among 181,536 cycles studied [8]. The incidence of POR decreased from 32% to 30% among cycles clinically assigned as DOR. Basal FSH ≥ 12 v/s clinical management assignment of DOR had a higher specificity (92.2% v/s 81.6%) and positive predictive value (38.3% v/s 30.9%) for predicting POR. They concluded that despite increasing DOR prevalence, the ability of clinical DOR to predict POR in the concurrent cycle worsened. More accurate markers of POR are needed to minimize patient anxiety, under and over diagnosis of POR. Possible explanations for increased DOR prevalence include advanced age, more diagnostic modalities and to explain suboptimal success rates to the patients labeled such.

## **Causes of DOR** [9–11]:

 Idiopathic

Chemotherapy

Radiotherapy

Genetic mutations like FMR

Smoking

Ovarian surgeries

Autoimmune

Mumps

Galactosemia

Tubal surgery

Idiopathic

Chemotherapy depletes primordial follicles in a dose and drug-dependent manner. Risk of toxicity during chemotherapy increases with age. Similarly, radiation affects ovaries depending on the dose, field and age of the patient [12]. Other causes of DOR include iatrogenic ovarian surgeries, uterine artery ligation, laparoscopic salpingectomy, genetic diseases (Turner’s galatosemia, Fragile X, FSH receptor and Inhibin B mutations), enzyme defects, mumps oophorihis, autoimmunity (Polyglandular syndrome, lymphocytic oophoritis, Addison’s disease, Hashimoto thyroiditis, celiac disease) and metabolic (Galactosemia)[13].

Idiopathic diminished ovarian reserve involves accelerated oocyte apoptosis. According to Barkers hypothesis, maternal endocrine disturbance during in utero life may result in DOR in the female fetus [14].

## Ovarian reserve estimation

Ovarian reserve is used to describe a woman’s reproductive potential by means of the quality and quantity of the oocytes her ovaries possess [15]. Ovarian reserve tests (ORT) aim at identifying women at risk of hypo or hyper response to ovarian stimulation, detecting reproductive lifespan and approximate menopausal timing and counseling and planning about a family, and individualizing management to optimize ovarian response whilst minimizing risks. Nevertheless, it is of utmost importance to understand that ORTs should not be used as a sole criterion to deny ART or other treatments to any patients. If ORTs indicate a diminished ovarian reserve, it means that pregnancy is less likely but not impossible. Though we use a battery of ORTs as a proxy for oocyte number, testing the oocyte quality or competence using these tests is poor.

Ideal ORT needs to be affordable, non-invasive, sensitive and specific, with minimal inter and intra cycle variability, with good sensitivity to detect the decline in ovarian reserve at a stage where timely interventions could lead to pregnancy [16]. ORTs should be offered to women at risk of DOR, not to the low-risk population.

ORT that are used as of now are biochemical, provocative and sonographic imaging of ovaries. The tests used for assessing ovarian reserve include basal day −3 follicle stimulating hormone (FSH, introduced in 1998). Clomiphene citrate challenge test (CCCT, 1989), gonadotropins releasing – hormone agonist stimulation test (GAST, 1989), Inhibin –B (1997), antral follicle count (AFC, 1997) and antimullerian hormone (AMH, 2002.)

The provocative tests like CCCT and GnRH-agonist stimulation test are literally out of practice now and FSH, AFC and AMH are being used extensively [17, 18].

## Basal FSH

This test is based on the negative feedback of FSH-pituitary secretion by ovarian factors like estradiol and inhibin B. Normal women have adequate quantities of ovarian hormones in early follicular phase, which maintains the FSH levels with normal range. However, women with depleting oocyte pool and diminished ovarian reserve will present with elevated basal FSH levels. Though basal FSH levels have significant inter and intra cycle variability, but when combined with basal estradiol, the sensitivity and specificity of basal FSH to test ovarian reserve is accentuated [19]. The specificity for using FSH to predict poor ovarian response is 45–100% (≤ 4 retrieved occytes). However the sensitivity is low 11–86% [19, 20]. FSH cut off values have been reset from 25 IU to 10 IU to detect expected poor ovarian response and failure to conceive [19].

## Basal estradiol

Early follicular estradiol (E_2_) levels are better used with basal FSH to assess ovarian reserve. Basal E_2_ has poor inter and intra cycle reliability. High basal E_2_ with normal FSH levels (>60–80 pg/ml) may indicate poor response and increased cycle cancellation rates [21].

## Antimullerian hormone (AMH)

AMH, a glycoprotein of the transforming growth factor – B (TGF – B), is produced by granulosa cells of small and large preantral and small antral follicles [22]. AMH levels peak at 25 years of age, gradually declining thereafter and reaching undetectable levels a few years before menopause [23–25]. AMH is secreted during early follicular stage by follicles up to 6 mm in size, is relatively gonadotropin–independent, and remain relatively constant within and between menstrual cycles [26–30]. Good evidence suggests that if significant fluctuations in AMH do occur, they are limited to younger women [31],, women with high AMH and aged women with low ovarian reserve [31, 32]. AMH is one of the best tests for ovarian reserve. It correlates very well with primordial follicle pool, ovarian response during ovarian stimulation and reliably predicts menopausal timing [33, 34]. AMH declines years before a rise in FSH is seen, thus proves to be a more sensitive biomarker of ovarian reserve [35, 36]. A systematic review of studies on women undergoing ovarian stimulation with gonadotropins by La Marca et al. (2010) identified low AMH cut-off points of 0.1–1.66 ng/mL to have sensitivities of 44–97% and specificities between 41 and 100% for predicting a poor ovarian response [22]. In addition, AMH correlates very strongly with the risk of ovarian hyper stimulation to gonadotropins, sensitivities range from 53 to 90.5% and specificities range from 70 to 94.4% using a cut – off of 3.36–5.0 ng/mL [22]. AMH is, however, a poor test for prediction of pregnancy and live births after ART, as is clearly concluded in 2 recent meta–analyses by Iliodromoti et al. [37] and Tal R, et al. [38] AMH should not be the sole criterion for denying fertility treatment. On the contrary, such women should be thoroughly counseled about possible low oocyte yield, high cycle cancellations and poor embryo quality.

AMH assessment is done using Enzyme –linked immunosorbent assay (ELISA) Gen II, Beckman Coulter Inc.; Brea, CA [39]. Newer automated AMH assay platforms are reported to offer better precision and sensitivity, faster results when compared to standard ELISA assays [40, 41]. Certain factors tend to influence the AMH values. These include PCOS (high), ovarian suppression with oral contraceptive pill (OCP) or GnRH agonists (Low), race and ethnicity, ovarian surgery, current smoking (low), and low vitamin D (low) [42–47]. Women with polycystic ovary morphology had significantly higher AMH levels than women in the control group. The prevalence of PCOS increased from 21% in the low-AMH (<4 ng/mL) group to 37% in the moderate-AMH (4–11 ng/mL) group and 80% in the high-AMH (>11 ng/mL) group [42]. Kallio S et al. studied the effects of oral contraceptives on serum levels of AMH and other hormones and concluded that antimullerian hormone (AMH), FSH, inhibin B, LH, and E2 levels had decreased significantly after 9 weeks of treatment [43].

Waylen AL et al. studies the effects of cigarette smoking on reproductive hormones (follicular serum concentrations of inhibin B hormone, FSH and AMH) in women of reproductive age and found a trend towards lower AMH and FSH concentrations in smokers, though it did not reach statistical significance [46]. A correlative and intervention study by Dennis NA, et al. to determine whether serum levels of AMH correlated with 25-hydroxyitamin D [25(OH)D)] status, and concluded that the change in AMH levels correlated with the magnitude of change in vitamin D levels and that cholecalciferol supplementation prevented seasonal AMH change [47].

## Antral follicle count (AFC)

AFC is the sum of follicles in both ovaries seen on ultrasound imaging during early follicular phase (Day 2–4). Antral follicles are defined as those measuring between 2 and 10 mm on a 2-dimensional plane. AFC is easy and quick to carry out, with good inter cycle and inter observer reliability [20], but has a compromised precision in women with weight extremes [20] and is dependent on the time of the cycle. A systematic review of ovarian reserve tests and IVF outcome by Broekman FJ et al. [20] concluded that low AFC is associated with poor ovarian response but has low predictability for pregnancy. The specificity of AFC for non- pregnancy predictor ranges between 64 and 98% but sensitivity stays low at 7–34% [20]. AFC also overestimates the actual number of FSH-sensitive follicles and oocyte yield, as it also measures atretic follicles of the same size [48].

## Ovarian volume and blood flow

Ovarian volume, calculated as D_1_ x D_2_ x D_3_ × 0.52 by measuring each ovary in three planes. This test has a limited value in testing ovarian reserve as it does not correlate well with pregnancy prediction [49]. In a meta- analysis where ovarian blood flow assessment was studies to predict IVF outcomes, it was concluded that this test is of unclear value [50].

## Which one is better – The debate

Leader B, et al. studied 5354 women to examine discordance between AMH and FSH results and found 1 in 5 women with discordant AMH and FSH values defined as AMH < 0.8 ng/ml (concerning) with FSH < 10 IU 0 L (reassuring) or AMH > 0.8 ng/ml (reassuring) with FSH ≥ 10 IU /L (concerning). AMH is more sensitive than FSH in diagnosing DOR, as was found in the study [51]. When ovarian reserve tests are discordant, it’s safe to go with an intermediate value between the two and administer an intermediate dose of gonadotropins stimulation [16] (Table 1)

**Table 1:** [16] Comparison between FSH, AMH and AFC

TEST	BASAL FSH	AFC	AMH
Cut–offs used for sensitivity and specificity	10 - 20	< 3–4 follicles, total	0.1–1.66 (ng/ml)
Sensitivity for Poor response (%)	11–86	9–73	44–97
Specificity for Poor response (%)	45–100	73–97	41–100
Sensitivity for Non Pregnancy (%)	3–65	7–34	19–66
Specificity for Non Pregnancy (%)	50–100	64–98	55–89

.

AMH < 0.5 ng/ml predicts an oocyte yield of ≤4 oocytes [22]. Patient should be counseled about an aggressive fertility treatment using microdose GnRH agonist flare with high starting gonadotropin dose or late luteal estradiol priming with or without late luteal presuppression antagonist with high starting gonadotropins dose [16, 22, 52].

AMH ≥ 1.0 ng/ml but ≤3.5 ng/ml predicts normal ovarian response and such women should undergo conventional GnRH agonist pr antagonist protocols [53–56].

## Whom to test?

Since nearly 10% of females undergo accelerated oocyte pool depletion, which leads to sub fertility and infertility early on, and with the present scenario of many women delaying childbearing, this issue may be of concern since it means many more women will end up in a poor ovarian response [57]. In the West, 25% women do not attempt pregnancy till 35 years of age [58]. Azhar E, et al. concluded that the knowledge of ovarian reserve would lead women to modify their reproductive decisions and make alternative decisions [59]. Women may become more well–versed with their reproductive lifespan and menopausal timing if offered ovarian reserve tests. Young cancer patients, who are planning to undergo gonadotoxic therapies, may especially benefit by ORTs.

Though there is a hot debate between the opponents and proponents of ovarian reserve testing in the general population, some advantages it offers includes identifying younger women with DOR who have a higher risk of early loss of fertility potential and advising them about their limited fertility option accordingly [60, 61].

## Preventing iatrogenic diminished ovarian reserve

Atabekoglu C et al. found that abdominal hysterectomy causes 30% more loss of ovarian function, although not statistically significant [62]. Hysterectomy was found to lead to early menopause and hysterectomy with unilateral oophorectomy to even earlier menopause in a prospective cohort study [63].

In a meta-analysis where the effect of surgery for endometriomas on ovarian reserve was studied, it was concluded that there’s a significant decline in AMH levels after ovarian cystectomy, with a mean weighted difference of 1.13 ng/ml. Endometriotic cyst excision was found to have a negative impact on ovarian reserve in terms of reduced AMH levels [64].

A recently published meta–analysis on changes in AMH levels after laparoscopic ovarian drilling (LOD) concluded that LOD markedly reduces AMH levels with PCOS who have normal or low AMH levels [65]. Salpingectomy was not shown to compromise ovarian reserve in the short- term.

Thus avoiding unnecessary surgeries and offering alternative therapies wherever indicated, will help in reducing the burden of iatrogenic diminished ovarian reserve.

In a recent meta-analysis by Mohamed AA et al., the mean age of menopause in current smokers is 1.74 ± 0.46 years earlier than non-smokers [66]. However, a dose-dependent effect was negligible [67].

Among young cancer patients, fertility preservation may be done by ovarian tissue cryopreservation (OTC), oocyte or embryo freezing prior to chemo/radiotherapy. Gonadotropin-releasing hormone analogue (GnRH – a) use has been found promising in preserving fertility if administered during chemotherapy [68]. Prior to pelvic radiotherapy, ovarian transposition can be done especially for pre-pubertal children.

Hormonal therapy for well-differentiated endometrial cancer and radical trachelectomy in early stage cervical cancer among young women may help preserve fertility to a great extent [69].

So far as the iatrogenic causes of DOR are concerned, strategies can be made to prevent damage to ovarian reserve. However, once ovarian reserve diminishes, unfortunately, not much can be done to replenish it.

## Social egg freezing- race against time or smoke and mirrors?

Human eggs have a definite decline in quality as well as quantity after 30 years of age, which plummets after 35. Social egg freezing is seen an insurance against age-related fertility decline. Social, personal, educational and financial pressures often lead women to delay starting a family until the late thirties, by which time the chance of getting pregnant is compromised by low fecundity rates and an increased risk of miscarriage if they become pregnant. In an internet-based survey on knowledge and attitudes of women towards social egg freezing in UK and Denmark, it was found that 83% women were aware about egg freezing option and 89% considered it acceptable for social reasons [70]. Characteristics significantly limited to the intention of egg freezing included being single, age under 35 years, childlessness and a history of infertility. The efficacy of the procedure was more important than its potential side effects and cost.

## The problem of poor ovarian response

Poor responders are a heterogeneous population as far as the pregnancy is concerned. Age and oocyte yield have a substantial effect on the pregnancy prospects in such patients [70]. The existing studies and research do not offer any clear – cut guidelines for clinical handling. A comprehensive review of trails published on “poor responders” by Papathanassiou A, et al. [71] concluded that a variety of definitions were used to define poor responders and a battery of interventions were done for head-to-head comparisons. This led to glaring discrepancies in the results of trials [71]. The existing criteria and definitions have not been able to sub classify POR patients who could benefit from particular interventions.

Comparing the various interventions for poor responders in IVF, it was concluded that the evidence to routinely support any particular intervention, either for pituitary down regulation, ovarian stimulation or use of adjutants, is insufficient [72]. The debate of oocyte quantity versus quality tips in favour of quality. Blastocyst euploidy rates drop drastically from 60% in women <35 years of age to 30% or lower in women aged 40–42 years, while age-related aneuploidy drastically rises [73].

Recently, the POSEIDON group (Patient - Oriented Strategies Encompassing IndividualizeD Oocyte Number) proposed a new classification of ART in patients with DOR or unexpected inappropriate ovarian response and four subgroups have been proposed based on quantitative as well as qualitative parameters which include

1. Age and expected anomaly rate,
2. Ovarian biomarkers like AFC and AMH, and
3. Ovarian response – if an earlier stimulation was performed [74].

**Table:** 

POSEIDON GROUP 1	POSEIDON GROUP 2
Young patients <35 years with adequate ovarian reserve (AFC ≥ 5; AMH ≥ 1.2 ng/ml) and with an unexpected poor or suboptimal ovarian response.	Older patients >35 years with adequate ovarian reserve (AFC ≥ 5; AMH ≥ 1.2 ng/ml) and with an unexpected poor or suboptimal ovarian response.
• Sub group 1a: < 4 oocytes* • Sub group 1b: 4–9 oocytes*	• Sub group 2a: < 4 oocytes retrieved* • Sub group 2b: 4–9 oocytes retrieved*
* Retrieved after standard stimulation.	* Retrieved after standard stimulation
POSEIDON GROUP 3	POSEIDON GROUP 4
Young patients <35 years with poor ovarian reserve pre – stimulation parameters (AFC < 5; AMH < 1.2 ng/ml)	Older patients ≥35 years with poor ovarian reserve pre – stimulation parameters (AFC < 5; AMH < 1.2 ng/ml)

The POSEIDON concept is expected to better classify women with low prognosis in Art, to help individualize their treatment to get at least one euploid embryo for transfer in each patient as a clear practical goal.

## Management

The management of DOR & POR can be a frustrating nightmare despite extensive studies and strategies. The strategies used are all aimed at a higher oocyte yield.

Various treatment regimens have been designed to manage the patients with diminished ovarian reserve and include high dose of gonadotropins, natural and modified natural cycles, estrogen priming, supplementation with LH, luteal antagonists and letrozole co-treatment use of adjuncts like androgens, growth hormone, melatonin and aspirin, oocyte donation and assisted hatching.

### Various protocols

## High dose gonadotropins

High dose of gonadotropins may not benefit the patient beyond a particular dose and may also increase the possibility of poor oocyte quality [75], patient discomfort and side effects. A retrospective analysis of 943 patients sub grouped according to the daily dose of gonadotropins (DD GN) was done for 1394 treatment cycles [76]. Group I received a “high dose” > 225 - ≤ 375 IU, Group II “very high dose” 376–450 IU, Group III “extremely high dose” 451–600 IU, controls received a DD GN of ≤225 IU. It was found that DD and total dose of GN negatively correlated with the oocyte yield, implantation, clinical pregnancy and live birth rates (25.9%, 14.6%, 11.4% and 4.7% in control, Groups I, II and III, respectively). Increasing the daily dose of GN higher than 450 IU or a total dose of 3000 IU / cycle is questionable. Furthermore, profound stimulation has a detrimental effect on luteal endocrine milieu and in turn affects endometrial receptivity [77, 78].

## Agonist and antagonist protocols

Comparing 2 ovarian stimulation protocols in 440 poor responders who had all failed to become pregnant during their first IVF cycle with long GnRH agonist protocol, Merviel P, et al. found that though the group treated with a contraceptive pill + flare up GnRH – agonist protocol had a higher number of embryos obtained, the implantation and ongoing pregnancy rates per transfer were the same between this group and the group with GnRH – antagonist protocol [79]. It was concluded that these two protocols resulted in similar implantation rates and that a customized ovarian stimulation regimen including mild ovarian stimulation, sequential IVF cycles, oocytes – embryos freeze all protocols and blastocyst transfers after screening may improve the outcome [79].

In a systematic review and meta – analysis of 12 studies, the effectiveness of GnRH antagonist in poor ovarian responders was evaluated, and it was found that stimulation period and gonadotropins dose, both were statistically significantly lower in the antagonist protocol compared to the long GnRH protocol. However, endometrial thicknesses, estrogen level on the day of HCG trigger and oocyte yield were lower in the antagonist group. The cycle cancellation and clinical pregnancy rates were not statistically significantly different between the two groups [80].

Song Y, et al. compared the effectiveness of GnRH antagonist / letrozole (A/L) protocol and the microdose GnRH agonist flare up protocol (MF) in a systematic review and meta- analysis [81]. The clinical pregnancy rate was significantly decreased with the A/L compared with MF protocol (RR 0.70; 95% CI: 0.57–0.86; *p* – 0.001) & the duration of gonadotropin stimulation was lower in the A/L group. There was no significant difference in cycle cancellation rates, oocyte yield and total dose of gonadotropins. Large –scale trials are needed to assess A/L protocol in view of the lower clinical pregnancy rate in this meta-analysis [82].

Various GnRH analogue protocols have been tried and tested in women with DOR. Late luteal GnRH agonist protocol may lead to excessive ovarian suppression in patients with DOR, which is not desirable as it may lead to reduced or even absent follicular response.

Various options tried in women with poor ovarian reserve include

1. Decreasing the length of suppression by using short, ultra-short, mini and micro dose flare up regimens,
2. Stopping or lowering the GnRH agonist after pituitary suppression, and
3. Using GnRH antagonist from mid-late follicular phase.

Despite widespread use of short and ultra short flare up regimens, none of the published studies have been able to establish any significant benefit on the clinical outcome in women with diminished ovarian response [81, 83, 84]. Two randomized trials failed to demonstrate any improvements in reproductive outcome when “GnRH agonist stopped protocol” was compared to the standard stimulation protocols [85, 86]. Similarly, in a recent meta –analysis, no statistically significant difference was found in clinical pregnancy rates, per cycle, duration of stimulation and total gonadotropin dose used, randomized between the GnRH agonist stopped protocol and the standard agonist protocol [87].

**GnRH antagonist** use is more patient friendly and decreases the amount and the number of days of gonadotropin stimulation. The introduction of GnRH antagonists is the key to development of milder and minimum stimulation protocols which allow for the initiation of the IVF treatment cycle in a normal menstrual cycle with an undisturbed recruitment of a cohort of follicles during the early follicular phase. This approach enables the endogenous inter-cycle FSH rise to be utilized rather than suppressed, resulting in a reduction of gonadotropins required. The treatment cycles are thus shorter and not associated with hypoestrogenic side effects related to GnRH agonist down-regulation, and reduce cancelation rates. Among the various advantages of using a GnRH antagonist in poor responders is the use of a newer long – acting hybrid gonadotropin, corifollitropin alfa that supports the follicle cohort for upto seven days. The corifollitropin rapidly increases serum FSH, which results in a significantly higher exposure of small antral follicles to high levels of FSH during early follicular phase and reduces the burden of daily injection [88] . Promising results were reported in a retrospective study on young poor responders using a combination of corifollitropin alfa with hp.-HMG in a GnRH antagonist protocol [89].

## Letrozole and clomiphene citrate co-treatment

In a recent retrospective analysis of 220 poor responders, the effects of different doses of letrozole (LZ) combined with gonadotropins (Gn) and high-dose gonadotropin stimulation in antagonist cycles were compared. Patients were divided into Group 1 (LZ 5 mg for 5 days sequentially overlapping with Gn cycles), group 2 (LZ 7.5 mg for 3 days sequentially with Gn cycles), and group 3 (high-dose Gn cycles) [90]. The amount of gonadotropin used in letrozole groups was lower with comparable pregnancy and live birth rates between the groups [90].

Group 1 had significantly higher early LH elevation rate on the HCG day than groups 2 and 3. Group 2 resulted in fewer improper LH surges and better outcomes than group 1.

Standard high dose gonadotropin – antagonist protocol (≥ 300 IU / day) was compared with a minimal stimulation protocol (150 IU / day) using overlapping with letrozole in antagonist cycle in poor responders, in a retrospective cohort study. The low dose protocol consisted of low dose 2.5 mg PO over 5 days, starting from cycle day 2. On day 4 of the cycle (day 3 of the letrozole treatment) overlapping low dose gonadotropins was started. The high-dose group received high levels of gonadotropins (≥300 IU/day) starting from day 2 of their cycle and throughout their short antagonist cycle. Clinical pregnancy rate was found to be significantly higher in the minimal stimulation group. In conclusion, the minimal stimulation IVF protocol was less expensive and resulted in a higher clinical pregnancy and live birth rate [91]. Since one protocol being compared involved letrozole and the other did not, it is not certain that the results were not related to the use of letrozole rather than high versus low dose gonadotropins.

## Minimal stimulation

A recent multi-center randomized non-infertility trial studied the pregnancy rates in mild ovarian stimulation strategy in women with poor ovarian reserve compared to those with conventional ovarian stimulation. Low – dose 150 IU FSH with antagonist was compared with high dose 450 IU HMG with long mid-luteal GnRH- agonist. The ongoing pregnancy rate in the minimal stimulation was 12.8% versus 13.6% for conventional stimulation group. The duration of ovarian stimulation and amount of gonadotropins used was significantly lower in the former group. The study concluded that high dosages of gonadotropins are not necessary in women with poor ovarian reserve [92].

A meta- analysis evaluated the efficiency of mild ovarian stimulation with and pregnancy outcome in poor ovarian responders and indicated that there was no significant difference for live birth and clinical pregnancy rates between conventional protocol of agonist and mild protocol of gonadotropins with CC and GnRH antagonist. It suggested that mild stimulation protocol with CC may give similar pregnancy outcomes in POR patients compared to the conventional stimulation protocol [93]. Oktem et al. found that severe poor responders who have previously failed to respond to microdose or antagonist protocol may benefit from CC plus HMG/Antagonist [94].

## The use of adjuvants

### **Androgen** (Dehydroepiandrosterone or testosterone)

Among the various adjuncts used to better the IVF outcome in poor responders are dehydroepiandrosterone (DHEA) and testosterone (T). DHEA and T are steroid hormones meant to increase conception rates by positively affecting follicular response to gonadotropin stimulation, in turn leading to better oocyte yield and pregnancy. Androgen also increases FSH receptor expression in granulose cells. Cochrane Database Systematic Review in 2015 indicated that pre-treatment with DHEA led to higher live birth and ongoing pregnancy rates compared to no treatment or placebo with an odds ratio of 1.88 and 95% CI. There was no evidence of a difference in miscarriage rates. Similarly, T compared to no / placebo treatment was associated with higher live birth rates with an odds ratio of 2.60, and 95% CI and no difference in the miscarriage rates. The authors concluded that in poor responders, pre-treatment with DHEA or T may improve live birth rates. In women with a 12% chance of live birth/ongoing pregnancy with placebo or no treatment, the live birth/ongoing pregnancy rate in women using DHEA was between 15% and 26%. In women with an 8% chance of live birth with placebo or no treatment, the live birth rate in women using testosterone was between 10% and 32%. To conclude, in poor responders undergoing ART, pre-treatment with DHEA or testosterone may be associated with improved live birth rates. The quality of evidence was moderate [95].

## Estradiol priming in luteal phase

Estradiol priming in the luteal phase with or without the simultaneous use of GnRH antagonist was found to decrease the risk of cycle cancellation and increase the chances of clinical pregnancy in a meta-analysis of 8 studies [96]. Luteal estradiol priming improves follicle synchronization. However, further studies are needed to establish the role of estradiol priming.

## Recombinant LH

Both LH and FSH are required for adequate ovarian estrogen biosynthesis and folliculogenesis. Theca cell derived androgen production controlled by LH is necessary for estrogen production by granulose cells. In mid-to-late follicular phase, FSH induces LH/hCG receptor expression in granulosa cells of large follicles [97]. In a recent meta-analysis of 40 studies, significantly more oocytes were retrieved and significantly higher clinical pregnancy rates were observed with recombinant – human FSH plus recombinant LH versus recombinant-FSH treatment alone in poor responders, suggesting that there’s an increase in the clinical pregnancy rates of 30% in poor responders with the addition of recombinant LH.

## Growth hormone (GH)

The use of GH is believed to modulate the action of FSH on granulose cells by up regulating the local synthesis of Insulin – like growth factor – I (IGF -1) [98]. Cycle cancellation rates and dose of gonadotropins were reduced in patients who received GH in a meta-analysis of 6 RCTs involving 169 patients [99]. Another meta-analysis involving 11 RCTs with 663 patients also reported significantly higher clinical pregnancy and live birth rates, oocyte yield, MII oocytes and E2 levels on the day of human chorionic gonadotropin (HCG). These two recent meta-analyses have suggested a significant improvement in clinical pregnancy and live birth rates, oocyte yield, mature oocytes with the use of GH [99, 100]. Large scale multi centric double blinded randomized controlled trials are needed to establish the efficacy and safety of GH beyond doubt in poor responders.

## Melatonin

Melatonin, a pineal gland hormone, regulates physiologic reproductive behavior and acts as a free radical scavenger [101]. Melatonin supplementation has been used to improve the outcome of IVF cycles in PCOS patients and women with DOR [102]. The mean estradiol level on the trigger day, mature oocyte yield and top quality embryos were higher among women who received melatonin. However, there was no difference on other ART outcomes between melatonin and no-melatonin groups [102].

## Aspirin

Good intra-ovarian blood flow is believed to improve the delivery of gonadotropic hormones for folliculogenesis and poor ovarian blood flow is linked to poor reserve [103, 104]. The use of aspirin in IVF has been a topic of debate. The conclusion of a meta-analysis in 2007 was that the clinical pregnancy rate per embryo transfer did not improve with low- dose aspirin; and the use of aspirin should not be routinely recommended [105].

## Oocyte accumulation

Novel vitrification technologies are being used to accumulate oocyte from several ovarian stimulation cycles, creating a similar situation as in normorespnder. Oocyte accumulation by vitrification followed by insemination yields comparable success in low responders [106]. It was possible to achieve higher live birth rates per patient and reduce dropouts with oocyte accumulation in a study by Cobo, et al. [106].

### Dual stimulation/double stimulation (follicular and luteal phase)

More recent evidence suggests that folliculogenesis occurs in a wave-like fashion through the menstrual cycle, and that there are multiple recruitment waves [107]. This has opened up a new horizon of opportunities to utilize ovarian stimulation in luteal phase following oocyte retrieval with follicular phase stimulation in the same cycle [108]. Typically, a luteal phase stimulation starts 2–7 days after oocyte retrieval in the same cycle. Either Gonadotropins or CC or Letrozole are used followed by trigger at lead follicle size of 18 mm. Embryo freezing is recommended in view of anticipated endometrial asynchrony. A randomized open-label pilot trial studies the luteal initiation of Gonadotropins in poor responders. The number of oocytes retrieved was similar during follicular and luteal phases. Serum estradiol levels, pregnancy and live birth rates did not differ between the two stimulation phases [109]^.^ In yet another study to evaluate the efficacy of double stimulation in poor responders undergoing mild ovarian stimulation it was showed that stimulation started in luteal phase could result in retrieval of more oocytes in a short period of time, which is important in poor responders [110]. In a more recent study, it was found that mean number of oocytes retrieved, mature oocytes and zygotes with two pronuclei was significantly higher for luteal stimulation collections in double stimulation cycles. The rate of clinical pregnancy and embryo implantation increased progressively from pure follicular phase embryos to mixed embryos to pure luteal phase embryos [111]. Larger double-blinded randomized studies are needed to establish the role of double stimulation beyond doubt.

## Oocyte donation

Egg donation may be the last efficient resort to offer hope to patients with diminished ovarian reserve and poor response. Though the pregnancy rates in women who use egg donation are at least as good as normo-responders, the decision is often difficult to make. Moreover, the facilities for egg donation, the acceptability and adequate counseling may not be available worldwide.

## Others

The newer yet-to-be established approaches in poor responders include ovarian transplantation, mitochondrial transfer and stem cell based neo-oogenesis [112, 113]. Some new data support the possibility to experimentally restore fertility in women with depleted ovarian reserve. Following the findings of germ line stem cells in adult mouse ovaries, several studies are trying to attempt isolating mitotic germ cells in adult human ovaries [114]. Virant – Klun et al. [114] tried to isolate the putative ovarian stem cells (OSCs) from the ovarian surface epithelium (OSE) in women with no naturally present oocytes and follicles. OSE scraping isolated small round cells, 2–4 μm in diameters, they were cultured and some oocyte like cells developed and reached 20 μm diameter and were termed “embryonic-like stem cells of the adult” and these oocyte – like cells underwent parthenogenetic activation to form blastocyst – like structures [114].

Experiments are being carried out towards development of artificial gametes from diploid somatic cells. Problems being faced are the inability of the somatic cells to reduce their chromosomes with the requisite fidelity and efficacy of a germ cell, which leads to high incidence of chromosomal abnormalities resulting from non- disjunction. More research is needed to determine whether these approaches are a realistic paradigm in the management of women with no follicles / eggs.

Our experience with diminished ovarian reserve and poor ovarian response [115] (Tables 2 and 3).

**Table 2:** ART cycles in patients with DOR

Age in years	25–30	31–35	36–40	>40
Number of cycles	9	17	60	31
Mean AMH ng/ml	1.09	0.99	0.74	0.4
Mean AFC	4	3	2	1
Pregnancy Rate %	33.33	29.4	21.82	12.9

**Table 3:** Cycle Characteristics & outcome (POSEIDON Criteria)

	Group1a	Group 1b	Group 2a	Group 2b	Group 3	Group 4
Number of patients	8	18	12	25	11	27
Age in years	<35	<35	>35	> 35	<35	≥ 35
Mean basal FSH mIU/ml	9.1	9.68	6.54	9.93	6.45	9.38
Mean basal E2	65	57.5	29	32.57	50	38.12
Mean AFC	8	9	5	6	4	2
Mean AMH ng/ml	3.1	2.82	1.42	2.19	0.64	0.71
Number of cycles	10	22	12	28	13	32
Total Gn dose IU	6675	7050	5850	6431	8250	8962
Mean number of oocytes retrieved	3	5	3	6	4	3
Mean number of mature oocytes	3	5	2	4	4	2
Fertilization Rate %	83.33	80	66.66	75	75	100
Cleavage Rate %	66.66	60	66.66	75	75	100
Pregnancy Rate %	40	45.45	25	39.28	15.38	37.50

## Conclusions

Despite a plethora of studies on diminished ovarian reserve and poor ovarian response, we are yet to reach a consensus and conclusion about their apt definition and management. Increasing the dose of gonadotropins, luteal phase gonadotropins, flare up agonist protocols, GnRH antagonists, supplementation with GH, Clomiphene citrate, letrozole, androgens and aspirin are among the various management strategies. We believe a thorough patient counseling and protocol personalization are the keys to optimize reproductive outcomes in such patients. Women with DOR should be appropriately counseled to undergo a rather- aggressive approach to achieve pregnancy before it is too late.

## Abbreviations

AL: Antagonist letrozole
AFC: Antral follicle count
AMH: Antimullerian hormone
ART: Assisted Reproductive Technologies
CC: Clomiphene citrate
CCCT: Clomiphene citrate challenge test
CI: Confidence interval
DD GN: Daily dose of gonadotropins
DHEA: Dehydroepiandrosterone
DOR: Diminished ovarian reserve
ELISA: Enzyme –linked immunosorbent assay
FSH: Follicle stimulating hormone
GAST: Gonadotropins releasing – hormone agonist stimulation test
GH: Growth Hormone
Gn: Gonadotrpins
GnRH – a: Gonadotropin-releasing hormone analogue
HCG: Human chorionic gonadotropin
HMG: Human Menopausal Gonadotropin
IGF −1: Insulin – like growth factor – I
IU: International Units
IVF: In Vitro Fertilization
LH: Luteinizing hormone
LOD: Laparoscopic ovarian drilling
OCP: Oral contraceptive pill
ORT: Ovarian reserve tests
OSCs: Ovarian stem cells
OSE: Ovarian surface epithelium
OTC: Ovarian tissue cryopreservation
POR: Poor ovarian response
POSEIDON group: (Patient - Oriented Strategies Encompassing IndividualizeD Oocyte Number)
SART: Society for Assisted Reproductive Technology
T: Testosterone
